# Schwangerschaftsabbrüche in Deutschland – Ergebnisse der Bundesstatistik

**DOI:** 10.1007/s00103-024-03994-3

**Published:** 2024-12-12

**Authors:** Heiko Pfaff

**Affiliations:** Referat „Pflege und weitere Sozialstatistiken“, Statistisches Bundesamt, Zweigstelle Bonn, Graurheindorfer Straße 198, 53117 Bonn, Deutschland

**Keywords:** Schwangerschaftsabbruch, Statistik, Statistisches Bundesamt, Schwangerschaftskonfliktgesetz, Meldestellen, Abortion, Statistics, Federal Statistical Office, Act on Assistance to Avoid and Cope with Conflicts in Pregnancy, Reporting healthcare providers

## Abstract

Nach einem Rückgang der Zahl der Schwangerschaftsabbrüche von ungefähr 130.000 jährlichen Abbrüchen in den Jahren von 1996 bis 2004 auf ca. 100.000 Fälle ab 2013/2014 gab es zuletzt wieder einen gewissen Anstieg auf 106.000 Abbrüche in 2023. Der Rückgang von 2004 bis 2013 war getragen von einer Abnahme der Zahl der Frauen im gebärfähigen Alter und auch von einer niedrigeren Wahrscheinlichkeit in den zentralen Altersgruppen, einen Abbruch durchzuführen. Bei einem Zeitvergleich der Ergebnisstrukturen ist bei der Art des Eingriffs insbesondere die stetige Entwicklung hin zum Einsatz von Mifegyne® auffällig. Bei der Dauer der abgebrochenen Schwangerschaft ist der Anteil der eher früh durchgeführten Abbrüche in den letzten Jahren gestiegen.

Zwischen den Bundesländern zeigen sich Unterschiede in den Strukturen. Die altersabhängige Wahrscheinlichkeit eines Abbruchs ist im Süden der Republik relativ niedrig, in den Stadtstaaten und in den neuen Ländern hingegen eher überdurchschnittlich. Weitere Unterschiede zwischen den Bundesländern hinsichtlich der genutzten Eingriffsart oder des Anbieters sind feststellbar. Auch der Anteil der in einem vom Wohnsitz der Frau abweichenden Bundesland durchgeführten Abbrüche variiert. Über die Angebotsseite kann die Anzahl der Meldestellen zusätzlich eine Basisinformation aus der Statistik bieten. Rechtliche Weiterentwicklungen insbesondere für eine verbesserte Regionalisierung der Daten – unter Achtung der statistischen Geheimhaltung – sind eingeleitet.

**Zusatzmaterial online:**

Zusätzliche Informationen sind in der Online-Version dieses Artikels (10.1007/s00103-024-03994-3) enthalten.

## Abstract (en)

After a decline in the number of annual abortions from around 130,000 in the years 1996 to 2004 to around 100,000 since 2013/14, there was recently a certain increase again to 106,000 abortions in 2023. The decline from 2004 to 2013 was driven by a decrease in the number of women of childbearing age and a lower probability of having an abortion in the key age groups. When comparing the result structures over time, the constant development towards the use of Mifegyne® is noticeable. Regarding the duration of the terminated pregnancy, the proportion of early abortions has increased in recent years.

There are differences in structures between the federal states. The probability of an abortion by age is relatively low in the south of the Republic but is above average in the city states and the new federal states. Further differences between the federal states can be identified with regard to the type of intervention used or the provider. The proportion of abortions carried out in another federal state also varies. The number of reporting healthcare providers can offer basic statistical information. Legal developments have been initiated, primarily for improved regionalization of data—while respecting statistical confidentiality.

## Einleitung

Der „Bericht der Kommission zur reproduktiven Selbstbestimmung und Fortpflanzungsmedizin“ zeigt das weiterhin hohe allgemeine Interesse am Thema Schwangerschaftsabbrüche sowie an der zugehörigen Datenlage. Die Kommission wurde durch den Koalitionsvertrag der Ampelregierung im Jahr 2021 initiiert, auch um Möglichkeiten einer Regulierung des Schwangerschaftsabbruchs außerhalb des Strafgesetzbuches zu prüfen. Zudem sei auf die vom Bundesministerium für Gesundheit (BMG) geförderte ELSA(Erfahrungen und Lebenslagen ungewollt Schwangerer – Angebote der Beratung und Versorgung)-Studie^1^ verwiesen, durch die wissenschaftlich basierte Erkenntnisse zu maßgeblichen Einflussfaktoren auf das Erleben und die Verarbeitung einer ungewollten Schwangerschaft, die Versorgungssituation und die Bedarfe betroffener Frauen in Deutschland gesammelt und ausgewertet wurden [1].

Das Bundesverfassungsgericht hatte bereits in seinem grundlegenden Urteil vom 28.05.1993 die weiter bestehende Notwendigkeit für eine Bundesstatistik zu den Schwangerschaftsabbrüchen betont [2]. Es erklärte die Aufhebung der Vorschrift über die Bundesstatistik über Schwangerschaftsabbrüche für grundgesetzwidrig: „Dem Gesetzgeber obliege es zu beobachten, ob die geltende gesetzliche Regelung tatsächlich einen angemessenen und wirksamen Schutz des werdenden Lebens bewirke. Diese Beobachtungspflicht, der eine Nachbesserungspflicht folgen könne, schließe es ein, dass der Gesetzgeber im Rahmen seiner Kompetenz dafür sorge, dass die für die Beurteilung der Wirkungen des Gesetzes notwendigen Daten planmäßig erhoben, gesammelt und ausgewertet würden“ [3]. Dieser Auftrag ist letztlich weiterhin eine wichtige Grundlage für die Ausgestaltung und die rechtliche Basis der bestehenden Statistik.

Im Folgenden werden zunächst der momentane rechtliche Rahmen und die Durchführung der Statistik knapp beschrieben. Danach werden die wichtigsten Ergebnisse für Deutschland im Jahr 2023 sowie die Entwicklung der Eckzahlen seit 1996 skizziert. Anschließend folgt eine Darstellung der zentralen Ergebnisstrukturen im Zeit- und im Bundesländervergleich. In einem Exkurs wird auf das Thema Meldestellen zur Statistik eingegangen. Abschließend wird ein Fazit gezogen sowie ein Ausblick auf sich abzeichnende Entwicklungen gegeben.

## Rechtlicher Rahmen und Durchführung

Die Bundesstatistik über Schwangerschaftsabbrüche wird in Deutschland auf der Grundlage des Schwangerschaftskonfliktgesetzes durchgeführt. In den Paragrafen 15 bis 18 ist festgelegt, dass die Daten zentral vom Statistischen Bundesamt in vierteljährlichem Abstand erhoben werden, welche Angaben zu erfragen sind und wer zur Statistik melden muss. Auskunftspflichtig sind dabei die Inhaberinnen und Inhaber der Arztpraxen und die Leitungen der Krankenhäuser, in denen Schwangerschaftsabbrüche vorgenommen werden. Die erhobenen Merkmale umfassen zum einen Angaben über ausgewählte Lebensumstände der betroffenen Frauen. Diese beinhalten deren Alter zum Zeitpunkt des Schwangerschaftsabbruchs, ihren Familienstand und die Zahl ihrer Kinder. Zu dem Abbruch werden die rechtliche Voraussetzung (Beratungsregelung oder nach Indikationsstellung), die Art des Eingriffs, die beobachteten Komplikationen und die Dauer der abgebrochenen Schwangerschaft erhoben. Erfasst wird auch, ob der Abbruch ambulant oder stationär im Krankenhaus erfolgte. Es wird außerdem das Bundesland erfragt, in dem der Schwangerschaftsabbruch vorgenommen wird, sowie das Bundesland, in dem die Schwangere wohnt bzw. ob sie im Ausland wohnt. Hingegen sind Frauen mit Wohnsitz in Deutschland, bei denen ein Schwangerschaftsabbruch im Ausland erfolgt, nicht Teil der Statistik.

Die Statistik wird seit 1996 auf dieser Basis grundsätzlich gut vergleichbar durchgeführt. Seit 1996 gab es nur Detailänderungen am Erhebungskonzept der Statistik.^2^ Zu den Erhebungen vor 1996 ist die Vergleichbarkeit der Daten hingegen deutlich eingeschränkt.^3^ Die Datenerhebung erfolgt über das gesicherte Online-Meldesystem im Statistischen Verbund (IDEV).

## Zentrale Ergebnisse für Deutschland 2023

Die Zahl der Schwangerschaftsabbrüche in Deutschland im Jahr 2023 lag bei rund 106.000 gemeldeten Fällen [4, 5].

Rund 65 % der Frauen, die im Jahr 2023 einen Schwangerschaftsabbruch durchführen ließen, waren zwischen 20 und 34 Jahren alt, gut 19 % waren im Alter zwischen 35 und 39 Jahren. Dies sind damit die am stärksten besetzten Altersgruppen (Tab. 1). 8 % der Frauen waren 40 Jahre und älter, 7 % waren jünger als 20 Jahre. 42 % der betroffenen Frauen hatten vor dem Schwangerschaftsabbruch noch kein Kind zur Welt gebracht.

**Tab. 1:** Schwangerschaftsabbrüche in Deutschland – Zeitreihe ab 2000 in Mehrjahresschritten (Strukturdaten der Schwangerschaftsabbruchstatistik, Statistisches Bundesamt)

Erhebungsmerkmal	2000	2005	2010	2015	2020	2023
Anzahl insgesamt	134.609	124.023	110.431	99.237	99.948	106.218
*Altersgruppen (Anteil in %)*						
Unter 15	0,4	0,5	0,4	0,3	0,3	0,3
15–17	4,3	5,3	3,7	3,0	2,4	2,4
18–19	6,8	7,4	6,8	5,3	4,5	4,7
20–24	21,2	23,6	24,6	20,8	19,1	18,9
25–29	21,7	21,4	23,0	24,5	22,6	22,4
30–34	22,6	18,4	19,4	22,0	24,4	23,6
35–39	16,6	16,1	14,5	16,5	18,7	19,3
40–44	5,9	6,8	7,1	6,8	7,4	7,8
45–49	0,5	0,5	0,6	0,7	0,6	0,7
50 und mehr	0,0	0,0	0,0	0,0	0,0	0,0
*Begründung des Abbruchs (Anteil in %)*						
Medizinische Indikation	2,7	2,6	2,8	3,9	3,8	3,8
Kriminologische Indikation	0,0	0,0	0,0	0,0	0,0	0,0
Beratungsregelung	97,3	97,4	97,2	96,1	96,2	96,2
*Art des Eingriffs (Anteil in %)*						
Curettage	11,2	10,5	10,5	13,3	12,1	9,2
Vakuumaspiration	82,6	79,1	71,9	64,4	54,9	48,1
Hysterotomie und Hysterektomie	0,0	0,0	0,0	0,0	0,0	0,0
Mifegyne	3,1	8,2	14,6	18,6	29,0	38,3
Medikamentöser Abbruch	3,1	2,2	2,7	3,0	3,4	3,7
Fetozid bei Mehrlingsschw.	n. e.	n. e.	0,0	0,1	0,1	0,0
Fetozid bei sonstigen Fällen	n. e.	n. e.	0,3	0,6	0,6	0,7
*Dauer der abgebrochenen Schwangerschaft (vollendete Wochen, Anteil in %)*						
Unter 7	n. e.	n. e.	37,4	35,8	41,2	45,5
7–8	n. e.	n. e.	35,4	36,2	34,2	32,6
9–11	n. e.	n. e.	24,9	25,2	21,7	18,9
12–21	n. e.	n. e.	1,9	2,2	2,2	2,3
22 und mehr	n. e.	n. e.	0,4	0,6	0,6	0,7
*Ort des Eingriffs (Anteil in %)*						
Gynäkologische Praxis	69,0	77,8	79,0	78,1	81,2	83,6
Krankenhaus (ambulant)	21,9	19,4	18,5	19,1	15,9	13,6
Krankenhaus (stationär)	9,1	2,8	2,6	2,8	2,9	2,8
*Anzahl der vorangegangenen Lebendgeburten (Anteil in %)*						
Keine	38,4	40,6	40,3	39,1	40,7	42,5
1	25,5	26,3	25,7	25,1	22,0	20,7
2	24,8	23,1	22,7	23,3	23,7	22,5
3	8,2	7,2	8,0	8,6	9,3	9,4
4	2,2	1,9	2,2	2,6	2,8	3,2
5 und mehr	1,0	0,9	1,1	1,3	1,5	1,8

*n.* *e.* nicht erhoben

Nach der Beratungsregelung wurden 96 % der im Jahr 2023 gemeldeten Schwangerschaftsabbrüche vorgenommen. Medizinisch begründete Indikationen lagen in 4 % der Fälle vor. Kriminologisch begründete Indikationen kommen entsprechend nur selten vor. Die meisten Abbrüche erfolgten nach 5 bis 6 vollendeten Wochen (34 %) sowie nach 7 bis 8 Wochen (33 %). Ab der 12. Schwangerschaftswoche wurden 3 % der Abbrüche durchgeführt. Im Zuge des Abbruchs beobachteten die Meldestellen in 2023 rund 300 Komplikationen. Dies betraf insbesondere Blutverlust und Nachblutungen (Tabelle Z1 im Onlinematerial).

Am häufigsten (48 %) wurde die Absaugmethode (Vakuumaspiration) beim Eingriff genutzt, bei 38 % der Abbrüche wurde das Mittel Mifegyne® verwendet. Die Eingriffe erfolgten überwiegend ambulant, davon rund 84 % in Arztpraxen beziehungsweise OP-Zentren und 14 % ambulant im Krankenhaus. Knapp 8 % der Eingriffe wurden in einem vom Wohnort der Frau abweichenden Bundesland durchgeführt.^4^

## Entwicklung der Zahl der Abbrüche seit 1996

**Abb. 1:**
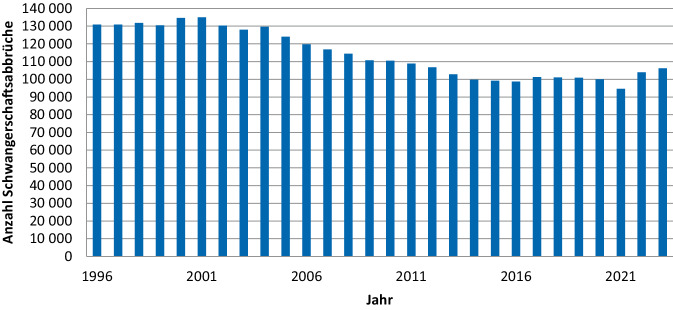
Anzahl der Schwangerschaftsabbrüche in Deutschland ab 1996. Eigene Abbildung (Quelle: Statistik der Schwangerschaftsabbrüche, Statistisches Bundesamt)

Die Zahl der jährlichen Abbrüche lag zwischen 1996 und 2004 grob überschlagen bei 130.000 Fällen (Abb. 1). Nach 2004 ist eine fortlaufende Abnahme festzustellen: So waren es im Jahr 2006 120.000 Schwangerschaftsabbrüche, 2010 dann rund 110.000. Ab 2013/2014 betrug die Anzahl ca. 100.000. Im zweiten Jahr der COVID-19-Pandemie 2021 fand dann ein stärkerer Rückgang auf rund 95.000 jährliche Abbrüche statt. 2022 und 2023 stieg die Zahl der Abbrüche auf 104.000 bzw. 106.000.^5^

Eine Ursache für den langfristigen Rückgang nach 2004 ist, dass die Zahl der Frauen im gebärfähigen Alter zurückging. So gab es Ende 2004 in Deutschland 19,6 Mio. Frauen im Alter von 15 bis 49 Jahren. 2013 betrug die Zahl hingegen 17,6 Mio. 2023 waren es 17,1 Mio. Insbesondere die starken Babyboom-Jahrgänge verließen sukzessive diese Altersphase.

**Abb. 2:**
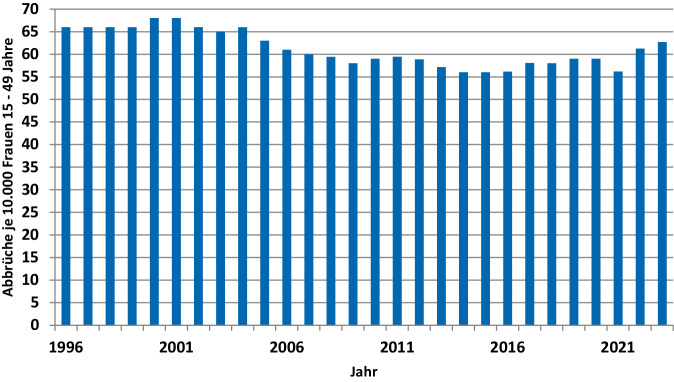
Anzahl der Schwangerschaftsabbrüche je 10.000 Frauen im Alter von 15 bis 49 Jahren in Deutschland ab 1996. Eigene Abbildung (Quelle: Statistik der Schwangerschaftsabbrüche, Bevölkerungsstatistik (2022), Statistisches Bundesamt)

Zudem nahm der Anteil der Schwangerschaftsabbrüche je 10.000 Frauen in der Altersgruppe 15 bis 49 Jahren ab (Abb. 2). Der Indikator lag z. B. im Jahr 2004 bei 66 und 2020 bei 59 und nun im Zuge der letzten Anstiege bei 63. Am niedrigsten fiel er in den Jahren 2014 bis 2016 sowie 2021 mit rund 56 aus. Der Indikator zeigt die Wahrscheinlichkeit in dieser Altersgruppe, einen Abbruch in dem jeweiligen Jahr durchzuführen. Die skizzierte Entwicklung des Indikators ist auch an der Zahl der Schwangerschaftsabbrüche bezogen auf die Geburten ablesbar. Dieser alternative Indikator ist aufgrund der teilweise größeren Schwankungen bei den Geburtenzahlen allerdings etwas volatiler.^6^

**Abb. 3:**
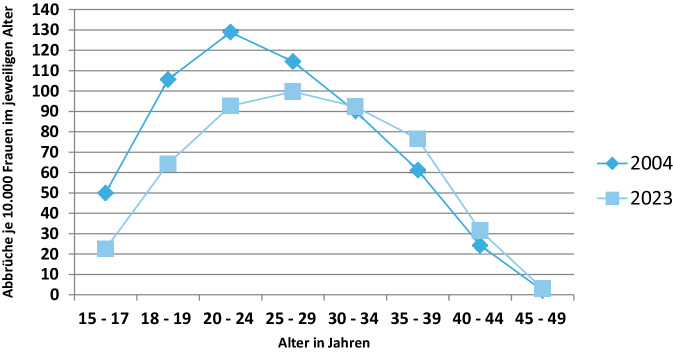
Anzahl der Schwangerschaftsabbrüche je 10.000 Frauen in den jeweiligen Altersgruppen in Deutschland 2004 und 2023. Eigene Abbildung (Quelle: Statistik der Schwangerschaftsabbrüche, Bevölkerungsstatistik (2022), Statistisches Bundesamt)

Noch deutlicher wird dieser rückläufige Effekt, wenn man die Altersgruppen weiter differenziert und die Anteile in den jeweiligen Altersgruppen über einen längeren Zeitraum vergleicht (2004 vs. 2023 siehe Abb. 3). Es zeigt sich eine deutliche Abnahme der altersspezifischen Abbruchwahrscheinlichkeiten insbesondere bei den unter 25-Jährigen – in diesem Alter werden also inzwischen seltener Abbrüche durchgeführt. Während der Wert z. B. im Jahr 2004 für die 20- bis 24-jährigen Frauen noch bei 129 lag, beträgt er inzwischen rund 93. In der Altersgruppe ab 35 Jahren zeigt sich im Langzeitvergleich hingegen – verstärkt bei den letzten Erhebungen – ein Anstieg. So lag bei den 35- bis 39-jährigen Frauen der Wert im Jahr 2004 bei 61. Inzwischen beträgt er in diesem Alter 76 Abbrüche je 10.000 Frauen.

Am relativ häufigsten erfolgten Abbrüche in 2023 im Alter von 25 bis 29 Jahren. Eher selten waren sie bei unter 18-jährigen und ab 40-jährigen Frauen (Abb. 3).

## Entwicklung der Ergebnisstrukturen im Zeitvergleich

Die Ergebnisstrukturen in Deutschland sind im Zeitvergleich zum Teil recht konstant. So betrug der Anteil der Abbrüche im Rahmen der Beratungsregelung im Jahr 2000 und 2010 gut 97 %. Heute sind es etwas mehr als 96 % (Tab. 1). Veränderungen zeigen sich durch die oben beschriebenen Entwicklungen in der Altersstruktur. Im Jahr 2005 waren 13 % der betroffenen Frauen unter 20 Jahre alt. Heute sind es 7 %. In dem Zeitraum hat der Anteil der Frauen ab 35 Jahren von 23 % auf 28 % zugenommen.

Auffallende Unterschiede gibt es im Zeitverlauf bei der Art des Eingriffs. Hier hat der Einsatz von Mifegyne® kontinuierlich an Bedeutung gewonnen. Der Anteil stieg von 3 % im Jahr 2000 über 15 % in 2010 auf heute 38 %. Parallel war der Anteil der Vakuumaspiration von 83 % im Jahr 2000 auf heute 48 % rückläufig.

Hinsichtlich des Zeitpunkts des Abbruchs zeigen sich auch Änderungen: Der Anteil der eher frühen Abbrüche vor der 7. vollendeten Woche hat zugenommen von 36 % (2015) auf nun 45 %. Der Anteil der Abbrüche ab der 12. vollendeten Woche liegt hingegen in den letzten Jahren recht konstant bei rund 3 %. Der Anteil der Abbrüche von der 7. bis zur 11. Woche ist entsprechend rückläufig.

Die stationär im Krankenhaus durchgeführten Abbrüche haben bereits Anfang des Jahrtausends relativ klar abgenommen. Dort wurden im Jahr 2000 noch 9 % aller Abbrüche durchgeführt. Nach 2003 sank der Anteil auf rund 3 % und ist seitdem relativ stabil.

## Vergleich nach Bundesländern

Hinsichtlich des Anteils der Schwangerschaftsabbrüche je 10.000 Frauen der Altersgruppe 15 bis 49 Jahren bestehen Unterschiede zwischen den Bundesländern (Tab. 2). Dieser Anteil ist – wie erwähnt – ein Indikator für die Wahrscheinlichkeit, in diesem Alter einen Abbruch in dem jeweiligen Jahr durchzuführen. Die niedrigsten Werte liegen im Süden der Republik in Bayern (47), Baden-Württemberg und Rheinland-Pfalz (jeweils 49). Der entsprechende Indikatorwert für Deutschland insgesamt beträgt 63 Abbrüche je 10.000 Frauen im gebärfähigen Alter. Die neuen Länder weisen überdurchschnittliche Werte zwischen 75 und 87 auf. In den Stadtstaaten Berlin (108) und Bremen (106) liegt zudem eine klar überdurchschnittliche Abbruchwahrscheinlichkeit vor. Der Wert für Hamburg (66) liegt 2023 nur etwas über dem Bundesdurchschnitt.

**Tab. 2:** Ausgewählte Indikatoren zur Struktur der Schwangerschaftsabbrüche nach Bundesländern 2023 (Quelle: Statistik der Schwangerschaftsabbrüche, Bevölkerungsstatistik (2022), Statistisches Bundesamt, ergänzende Berechnungen)

	Indikator				
Bundesland	Abbrüche je 10.000 Frauen im Alter von 15 bis 49 Jahren	Anteil der Abbrüche in %			
		Mit der Eingriffsart Mifegyne®	Ambulant im Krankenhaus	In einem vom Wohnsitz der Frau abweichenden Bundesland^a^ „Exporte“	Von Frauen mit Wohnsitz in einem anderen Bundesland^a^ „Importe“
Baden-Württemberg	49	41	9	11	3
Bayern	47	28	3	7	4
Berlin	108	54	3	1	5
Brandenburg	75	42	50	16	2
Bremen	106	32	2	9	42
Hamburg	66	44	9	3	19
Hessen	68	34	7	3	10
Mecklenburg-Vorpommern	80	50	43	4	2
Niedersachsen	56	34	24	21	7
Nordrhein-Westfalen	63	37	4	2	3
Rheinland-Pfalz	49	17	8	39	27
Saarland	78	13	4	1	33
Sachsen	76	47	37	1	6
Sachsen-Anhalt	87	36	38	6	3
Schleswig-Holstein	62	57	35	5	2
Thüringen	82	40	48	7	11
*Deutschland gesamt*	*63*	*38*	*14*	*8*	*8*

^a^Ohne Frauen mit Wohnsitz im Ausland

Auch in den weiteren Strukturen zeigen sich beispielsweise folgende deutliche Unterschiede: Der Anteil der mit Mifegyne® durchgeführten Abbrüche variiert deutlich zwischen den Ländern. Die höchsten Anteile liegen hierbei in Schleswig-Holstein (57 %) und Berlin (54 %). Die niedrigsten im Saarland (13 %) und Rheinland-Pfalz (17 %). Im bundesweiten Mittel beträgt der Anteil 38 %. Der Anteil der in einem vom Wohnsitz der Frau abweichenden Bundesland durchgeführten Abbrüche ist ebenfalls divergent: Hier finden sich die höchsten Werte in Rheinland-Pfalz (39 %) und Niedersachsen (21 %). Den niedrigsten „Exportanteil“^7^ haben Berlin, Saarland und Sachsen mit je 1 %. Der bundesweite Vergleichswert liegt bei 8 %. Den höchsten „Importanteil“ bezogen auf die im Bundesland durchgeführten Eingriffe haben Bremen (42 %) und das Saarland (33 %), den niedrigsten haben mit jeweils 2 % Schleswig-Holstein, Brandenburg und Mecklenburg-Vorpommern.^8^

Auch die Bedeutung der ambulant im Krankenhaus durchgeführten Abbrüche variiert klar zwischen den Ländern. In Brandenburg (50 %) und Thüringen (48 %) finden sich die Höchstwerte, die niedrigsten in Bremen (2 %), Bayern und Berlin (jeweils 3 %). Dort haben die Praxen und OP-Zentren eine deutlich stärkere Bedeutung für die Versorgung.

## Exkurs: Meldestellen zur Statistik

Wie bereits beschrieben stehen die durchgeführten Schwangerschaftsabbrüche sowie die betroffenen Frauen im Fokus der Statistik nach dem Schwangerschaftskonfliktgesetz. Nach dem Gesetz werden hierzu Kliniken und Arztpraxen befragt, in denen grundsätzlich Abbrüche vorgenommen werden (sogenannte Meldestellen). Die Adresse der Meldestelle ist rechtlich ein Hilfsmerkmal. Nach § 10 BStatG dienen die Hilfsmerkmale grundsätzlich der technischen Durchführung von Bundesstatistiken. Sie sind demnach zu unterscheiden von Erhebungsmerkmalen, die zur statistischen Verwendung bestimmt sind. Für die Hilfsmerkmale bestehen engere Löschvorgaben und Verwendungsgrenzen.^9^

Seit dem 4. Quartal 2018 wird aufgrund verstärkter Nachfragen der Statistiknutzenden aus den Adressangaben der Meldestellen (Praxen und Krankenhäuser) deren Anzahl systematisch durch das Statistische Bundesamt ermittelt. Bundesweit betrug sie dabei zuletzt rund 1100 Stellen. Die Zahl der Meldestellen wird für die einzelnen Bundesländer nachgewiesen (Tab. 3). Eine tiefere Regionalisierung der Meldestellen ist nicht verfügbar. Hier bestehen bis dato rechtliche Grenzen durch die grundsätzliche Vorgabe für die Erfassung der Erhebungsmerkmale bis auf Ebene der Bundesländer. Mögliche Weiterentwicklungen der Regionalisierung im Rahmen der bestehenden Erhebung sind Teil des nächsten Abschnittes „Fazit und Ausblick“.

**Tab. 3:** Anzahl der Meldestellen^a^ zur Schwangerschaftsabbruchstatistik in Deutschland 2018–2023 (Quelle: Statistik der Schwangerschaftsabbrüche, Statistisches Bundesamt)

Bundesland	2018	2019	2020	2021	2022	2023
Baden-Württemberg	93	94	96	94	96	98
Bayern	95	93	93	89	88	83
Berlin	134	141	136	134	135	134
Brandenburg	53	50	47	48	47	50
Bremen	13	12	16	18	21	23
Hamburg	56	55	51	50	51	51
Hessen	81	81	75	74	74	73
Mecklenburg-Vorpommern	48	49	44	45	46	48
Niedersachsen	116	113	114	115	109	108
Nordrhein-Westfalen	164	159	148	144	152	151
Rheinland-Pfalz	29	29	27	26	27	27
Saarland	11	10	9	9	9	9
Sachsen	111	110	107	101	106	106
Sachsen-Anhalt	49	48	47	47	47	46
Schleswig-Holstein	66	65	61	61	58	58
Thüringen	41	40	38	37	39	39
*Insgesamt*	*1160*	*1149*	*1109*	*1092*	*1105*	*1104*

^a^Die Anzahl der Meldestellen wird anhand der befragten Meldestellen je Quartal ermittelt (über die Adressen der jeweils Auskunftspflichtigen). Sie lässt keine Rückschlüsse auf die genaue Zahl der Arztpraxen bzw. Kliniken mit Abbrüchen zu. Zum einen sind auch Meldestellen mit Fehlmeldungen (keine Abbrüche im Quartal) enthalten, zum anderen melden zentrale ambulante OP-Praxen hier z. B. für mehrere Arztpraxen mit

Faktisch sind zudem aufgrund der relativ geringen Zahl der Meldestellen die regionalen Auswertungsmöglichkeiten sowie differenziertere Darstellungen der Meldestellen wegen der dann verstärkt auftretenden Geheimhaltungs- und Zuordnungsrisiken der Datenmeldungen deutlich eingeschränkt (siehe auch „Fazit und Ausblick“).

Auswertungen zur Gesamtzahl der Meldestellen wurden vor 2018 nicht systematisch erstellt. Nachträgliche Auswertungen sind nicht möglich, da nur die aktuell zur Befragung notwendigen Meldestellen (mit den Adressen) weitergeführt werden dürfen. Es liegen somit kaum historische Angaben zu den Meldestellen vor. Aus älterem Schriftverkehr ist bekannt, dass es im Jahr 1999 rund 1.650 Meldestellen und im Jahr 2003 – trotz einer ähnlichen Anzahl an jährlichen Schwangerschaftsabbrüchen – etwa 2.050 Meldestellen in Deutschland gab. Eine regionale Differenzierung liegt für diese Jahre nicht vor.

Die Anzahl der Meldestellen schwankt von Quartal zu Quartal und lässt auch keine Rückschlüsse auf die genaue Zahl der Arztpraxen beziehungsweise Kliniken mit Abbrüchen zu. Zum einen sind auch Meldestellen mit Fehlanzeigen (keine Abbrüche im Quartal) enthalten, zum anderen melden zentrale ambulante OP-Praxen zum Beispiel für mehrere Arztpraxen mit. Diese Einschränkungen müssen bei Aussagen, Interpretationen und Zeitvergleichen beachtet werden. Letztendlich bietet die Auswertung zu den Meldestellen somit eine eher grobe Orientierung. Die Angabe ist aber zumindest die beste Information, die durch diese Routineerhebung zusätzlich über die Anbieterseite gewonnen werden kann. Sie weist – wie angeführt – im langfristigen Vergleich eine rückläufige Entwicklung der Anzahl der Meldestellen aus. Auf eine tiefere Interpretation der Daten wird an dieser Stelle verzichtet. Zum einen variiert die Größe der Meldestellen erfahrungsgemäß und eine Ableitung stabiler Aussagen ist somit erschwert. Auch sind sie – vor dem Hintergrund der beschriebenen Einschränkungen der Aussagekraft, der zum Teil niedrigen Fallzahlen, möglicher Zuordnungsrisiken und rechtlicher Grenzen – bis dato kein vertiefter Bestandteil der Routineberichterstattung im bestehenden rechtlichen Rahmen.

## Fazit und Ausblick

Nach einem Rückgang der Zahl der Schwangerschaftsabbrüche von ungefähr 130.000 jährlichen Abbrüchen in den Jahren von 1996 bis 2004 auf ca. 100.000 Fällen ab 2013/2014 gab es zuletzt wieder einen gewissen Anstieg auf 106.000 Abbrüche in 2023. Der Rückgang von 2004 bis 2013 war getragen von einer Abnahme der Zahl der Frauen im gebärfähigen Alter und auch von einer niedrigeren Wahrscheinlichkeit in den zentralen Altersgruppen, einen Abbruch durchzuführen. Bei einem Zeitvergleich der Ergebnisstrukturen ist bei der Art des Eingriffs insbesondere die stetige Entwicklung hin zum Einsatz von Mifegyne® auffällig. Bei der Dauer der abgebrochenen Schwangerschaft ist der Anteil der eher früh durchgeführten Abbrüche in den letzten Jahren gestiegen.

Zwischen den Bundesländern zeigen sich Unterschiede in den Strukturen. Die Abbruchwahrscheinlichkeit nach Alter ist im Süden der Republik relativ niedrig, in den Stadtstaaten und in den neuen Ländern hingegen eher überdurchschnittlich. Weitere Unterschiede zwischen den Bundesländern hinsichtlich der genutzten Eingriffsart oder des Anbieters sind feststellbar. Auch der Anteil der in einem vom Wohnsitz der Frau abweichenden Bundesland durchgeführten Abbrüche variiert. Über die Angebotsseite kann die Zahl der Meldestellen zusätzlich eine Basisinformation aus der Statistik bieten.

Die Ursachen für die Entwicklungen sind allein anhand der Bundesstatistik nicht erklärbar. Eine Erhebung der Gründe für einen Abbruch ist im Rahmen dieser amtlichen Befragung mit Auskunftspflicht der Praxen und Kliniken kaum effizient umsetzbar. Hier erscheinen Stichprobenbefragungen außerhalb der amtlichen Statistik, die auch die betroffenen Frauen direkt einbeziehen, eher zielführend.

Momentan sieht ein Gesetzesentwurf unter anderem eine gezielte Ergänzung der Statistik vor [7–9]. Die vorgesehenen inhaltlichen Änderungen sollen einem besseren Überblick über die Versorgungssituation in den Bundesländern dienen. Bisher werden die Daten zu den Schwangerschaftsabbrüchen entsprechend der Rechtsgrundlage – wie dargestellt – standardmäßig nur auf Bundes- und Länderebene ausgewertet. Zukünftig soll die Bundesstatistik mit der anvisierten Änderung des Schwangerschaftskonfliktgesetzes jährlich auch Angaben über die regionale Verteilung der Schwangerschaftsabbrüche unterhalb der Länderebene ausweisen. Zur effizienten regionalen Zuordnung darf dann die Adresse der Meldestelle ausdrücklich genutzt werden. Durch diese Ausgestaltung könnten durch das neue Gesetz zusätzliche statistische Informationen gewonnen werden, ohne eine weitere Belastung für die Berichtsstellen zu erzeugen.

Bei den Auswertungen ist weiterhin die Pflicht zur statistischen Geheimhaltung zu wahren. Betroffen hiervon dürften insbesondere ländliche Regionen mit kleineren Fallzahlen bei den Meldestellen sein. In solchen Fällen sind die Ergebnisse von Kreisen und kreisfreien Städten entsprechend der Gesetzesbegründung regional zusammenzufassen. So können etwa 2 Kreise oder eine kreisfreie Stadt mit einem angrenzenden Kreis oder mehreren umliegenden Kreisen zusammengefasst dargestellt werden oder eine Zuordnung der Kreise nach bestehenden Raumordnungsregionen verwendet werden. Beim Ausweis der einzelnen Merkmale erfolgt zusätzlich eine Geheimhaltung mit den üblichen statistischen Verfahren des Statistischen Bundesamtes. Ein adäquates Auswertungssystem befindet sich momentan im Aufbau und soll möglichst erstmals rückwirkend für die Erhebung 2023 eingesetzt werden.

Nach den angestrebten rechtlichen Vorgaben sollen außerdem die Meldestellen künftig jährlich auf Bundes- und Länderebene nach Größenklassen gestaffelt dargestellt werden, um das Bild zur Versorgungslage zu verbessern. Zusätzlich kann das Statistische Bundesamt die Zahl der auf Ebene der Kreise und kreisfreien Städte bestehenden Meldestellen veröffentlichen, soweit dies vor dem Hintergrund der Geheimhaltungs- und Zuordnungsthematik und der Fallzahlen sinnvoll umsetzbar ist.

Der Gesetzesentwurf sieht eine weitere Anpassung vor. Für eine qualitativ gesicherte Erhebung ist eine zielgerichtete Pflege des Berichtskreises der Statistik notwendig: Die im Statistischen Bundesamt vorliegenden Anschriften der Einrichtungen müssen als Basis für eine erfolgreiche Erhebung vollständig und korrekt sein. Um die Pflege der Anschriften zu vereinfachen, sollen weitere, auch landesspezifische Quellen mit Adressangaben zu den Einrichtungen vom Statistischen Bundesamt herangezogen werden können. Dies kann etwa bei Behörden der Fall sein, die nach einem landesspezifischen Verfahren für die Freigabe bestimmter Medikamente an medizinische Einrichtungen zuständig sind. Dort müssten auch aktuelle Adressen über die Einrichtungen vorliegen. Gleiches gilt für Landeskrankenhausgesellschaften und kassenärztliche Vereinigungen. Die Pflicht zur Übermittlung an das Statistische Bundesamt auf Anforderung greift aber nur, wenn aufgrund der bestehenden Zuständigkeit Anschriften über Einrichtungen, in denen Schwangerschaftsabbrüche vorgenommen werden, bereits vorliegen. Somit sollen bis dato zusätzlich durchgeführte Befragungen potenzieller Meldestellen und Abstimmungen zur Pflege des Berichtskreises reduziert oder möglichst vermieden werden.

Die dargestellten Gesetzesanpassungen können basierend auf der bestehenden Erhebung insgesamt zu Verbesserungen der Statistik im Detail führen, ohne eine Mehrbelastung der meldenden Stellen zu erzeugen.

## Supplementary Information

Onlinetabelle Z1: Schwangerschaftsabbrüche 2023 nach Komplikationen und Art des Eingriffs

## Footnotes

1 Zu ELSA bietet eine Homepage umfassende Informationen: https://elsa-studie.de/, zugegriffen: 17.05.2024.

2 Seit 2010: Erfassung der Schwangerschaftsdauer bis zum Abbruch ausdrücklich nach vollendeten Wochen und Erfassung der Fetozide als Eingriffsart. Seit 2018 wird zudem die beim Eingriff verwendete Anästhesie nicht mehr erhoben.

3 Im früheren Bundesgebiet – und ab 1993 in ganz Deutschland – erfolgte die Meldung bis Ende 1995 auf einem Erhebungsbogen, der ohne Adressangabe der meldenden Stelle abgegeben werden konnte. Bis 1995 ist von einer erheblichen Untererfassung auszugehen. Erst ab 1996 besteht die Pflicht, zur Datenmeldung als ergänzendes Hilfsmerkmal Name und Anschrift der Einrichtung anzugeben. Seitdem kann die Einhaltung der Berichtspflicht kontrolliert werden.

4 Anteilsberechnung hier ohne die Abbrüche der 456 Frauen mit Wohnsitz im Ausland.

5 Umfangreiche Zeitreihen bis auf Ebene der Bundesländer siehe https://www.gbe-bund.de/gbe/isgbe.startseite, zugegriffen: 21.05.2024.

6 Die Werte sind z. B. in der in Fußnote 5 erwähnten Datenbank der GBE abrufbar. Eine strukturelle Beschreibung bietet auch [6].

7 Die eher ökonomisch geprägten Begriffe Export und Import sollen hier einem intuitiven Verständnis dienen.

8 In Brandenburg finden hingegen relativ viele Schwangerschaftsabbrüche von Frauen mit Wohnsitz im Ausland statt. 2023 waren es rund 280 der insgesamt 460 Ausländerinnen.

9 Die Darstellung beruht auf der entsprechenden Internetseite des Statistischen Bundesamtes: https://www.destatis.de/DE/Themen/Gesellschaft-Umwelt/Gesundheit/Schwangerschaftsabbrueche/aktuell-meldestellen.html bzw. https://www.destatis.de/DE/Themen/Gesellschaft-Umwelt/Gesundheit/Schwangerschaftsabbrueche/_inhalt.html, zugegriffen jeweils: 24.05.2024.
