# Supplementary material for: Abortions in Germany—results of federal statistics
Source: Bundesgesundheitsblatt Gesundheitsforschung Gesundheitsschutz. 2024 Dec 12;68(1):3–10. [Article in German] doi: 10.1007/s00103-024-03994-3 (PMC11732936; doi:10.1007/s00103-024-03994-3)
Supplement: Supplementary file 1 — Onlinetabelle Z1: Schwangerschaftsabbrüche 2023 nach Komplikationen und Art des Eingriffs [file 103_2024_3994_MOESM1_ESM.pdf]

Onlinetabelle Z1: Schwangerschaftsabbrüche 2023 nach Komplikationen und Art des Eingriffs

Art des Eingriffs	Insgesamt	Komplikationen													
		mit (zusammen)	Cervixriss	Uterus-perforation	Blutverlust	Blutüber-tragung	Nach-blutungen	Allgemein-infektionen	Fieber über 38ºC	Salpingitis	Parametritis	Thrombose/ Embolie	Narkose-zwischenfall	zum Tode führende Kompl.	sonstige Kompl.
	Anzahl	%													
Insgesamt..	106.218	0,3	0,0	0,0	0,1	0,0	0,1	0,0	0,0	-	0,0	-	0,0	-	0,1
Curettage..	9.784	0,2	0,0	0,0	0,1	-	0,0	0,0	0,0	-	-	-	-	-	0,1
Vakuumaspiration.....................................	51.084	0,2	0,0	0,0	0,1	0,0	0,0	0,0	0,0	-	0,0	-	0,0	-	0,0
Hysterotomie / Hysterektomie....................	1	-	-	-	-	-	-	-	-	-	-	-	-	-	-
Mifegyne®/ Mifepriston..............................	40.659	0,3	-	0,0	0,0	-	0,1	0,0	0,0	-	-	-	-	-	0,2
Medikamentöser Abbruch.........................	3.895	1,6	0,0	-	0,4	0,2	0,3	0,1	0,1	-	0,0	-	-	-	0,6
Fetozid bei Mehrlingsschwangerschaften...	37	2,7	-	-	-	-	-	-	2,7	-	-	-	-	-	-
Fetozid bei sonstigen Fällen	758	2,5	-	-	0,7	-	0,3	0,1	0,1	-	-	-	-	-	1,3

Statistisches Bundesamt - Statistik der Schwangerschaftsabbrüche

Hinweis:
„Mifegyne®/ Mifepriston“ beinhaltet Schwangerschaftsabbrüche entsprechend der Zulassung bis 6+6 Schwangerschaftswoche p.c.. Sie werden so getrennt von anderen medikamentösen Abbrüchen erfasst.
„Medikamentöser Abbruch“ umfasst die Wirkstoffe Prostaglandine oder Prostaglandin-Analoga mit oder ohne Mifepriston nach 6+6 SSW p.c.. Die Verwendung des Arzneimittels Ethacridin (Rivanol®) über die Cervix ist hier z. B. ebenfalls zugeordnet.
Werden mehrere Arten des Eingriffs (Schwangerschaftsabbruchmethoden) angewandt, ist diejenige anzugeben, die den Schwangerschaftsabbruch bewirkt hat.
